# Interpersonal Coordination and Individual Organization Combined with Shared Phenomenological Experience in Rowing Performance: Two Case Studies

**DOI:** 10.3389/fpsyg.2017.00075

**Published:** 2017-01-30

**Authors:** Ludovic Seifert, Julien Lardy, Jérôme Bourbousson, David Adé, Antoine Nordez, Régis Thouvarecq, Jacques Saury

**Affiliations:** ^1^Centre d'Etudes des Transformations des Activités Physiques et Sportives (CETAPS) - EA 3832, University of Rouen NormandyMont Saint Aignan, France; ^2^Laboratory “Movement, Interactions, Performance” (EA 4334), Faculty of Sport Sciences, University of NantesNantes, France

**Keywords:** ecological dynamics, perturbation, variability, phenomenology, experience

## Abstract

The principal aim of this study was to examine the impact of variability in interpersonal coordination and individual organization on rowing performance. The second aim was to analyze crew phenomenology in order to understand how rowers experience their joint actions when coping with constraints emerging from the race. We conducted a descriptive and exploratory study of two coxless pair crews during a 3000-m rowing race against the clock. As the investigation was performed in an ecological context, we postulated that our understanding of the behavioral dynamics of interpersonal coordination and individual organization and the variability in performance would be enriched through the analysis of crew phenomenology. The behavioral dynamics of individual organization were assessed at kinematic and kinetic levels, and interpersonal coordination was examined by computing the relative phase between oar angles and oar forces and the difference in the oar force impulse of the two rowers. The inter-cycle variability of the behavioral dynamics of one international and one national crew was evaluated by computing the root mean square and the Cauchy index. Inter-cycle variability was considered significantly high when the behavioral and performance data for each cycle were outside of the confidence interval. Crew phenomenology was characterized on the basis of self-confrontation interviews and the rowers' concerns were then analyzed according to course-of-action methodology to identify the shared experiences. Our findings showed that greater behavioral variability could be either “*perturbing*” or “*functional*” depending on its impact on performance (boat velocity); the rowers experienced it as sometimes meaningful and sometimes meaningless; and their experiences were similar or diverging. By combining phenomenological and behavioral data, we explain how constraints not manipulated by an experimenter but emerging from the ecological context of a race can be associated with functional adaptations or perturbations of the interpersonal coordination.

## Introduction

Interpersonal coordination means that the movements of at least two individuals are coupled. As observed in team sports, individuals can engage in cooperative (within team) and/or competitive (between teams) relationships, which influence the dynamics of the interpersonal coordination to reach the task-goal (Vilar et al., 2012; Passos et al., 2016). Rowing crews offer an interesting context for studying cooperative relationships because the rowers need to coordinate their action throughout the race and constantly adjust to each other (Hill, 2002; Baudouin and Hawkins, 2004; Sève et al., 2013; de Poel et al., 2016). This was shown, for example, by analyzing the within-crew coordination of force patterns, particularly by computing the area under the force–time curve differences and the force–time shape differences (i.e., to estimate the movement pattern) (Hill, 2002). In particular, Hill (2002) suggested that the kinesthetic perception of force–time shape differences is easier than perceiving area under the force–time curve differences when rowers regulate their coordination.

The two rowers of a coxless pair crew have a cooperative relationship, but it is of a certain type: leader–follower. When the boat has more than one rower, the rower closest to the stern of the boat is referred to as the “stroke,” whereas the rower at the opposite end of the boat is referred to as the “bow.” The “stroke” rower is the leader, because he/she is supposed to set the stroke frequency for the rest of the crew to follow (Nolte, 2011). Therefore, although rowing is a cooperative endeavor, it is expected that the stroke rower will drive or lead the crew, while the bow rower is driven or follows the stroke's lead. Although the status of leader and follower is given in advance in the crew, it could be expected that any behavioral fluctuations of one rower (for personal reasons such as fatigue or for environmental reasons such as wind, waves, other boats or changes in the river pathway) or of the boat will disturb the stability of the system organization (both at the interpersonal coordination and boat velocity levels). In this case, it cannot always be assumed that the stroke rower alone will restore the stability of the interpersonal coordination and maintain high boat velocity. Among the parameters used to describe rowing performance and explain high boat velocity, the stroke frequency and the variations in boat velocity are important (Hill and Fahrig, 2009; Rauter et al., 2012). As propulsion alternates with oar recovery, the variations in boat velocity cannot be avoided, which led Hill and Fahrig (2009) to suggest that variations in boat velocity can cost as much as an additional 5 s in a 2000-m race compared with a boat hypothetically moving with constant velocity. Therefore, these authors noted that “*a slight reduction of velocity fluctuations may be achieved by a moderate reduction of stroke frequency compensated by an increased force output for each stroke”* (p. 593), which seems reachable only by elite rowers (Hill and Fahrig, 2009). As this biomechanical aspect is among the most technical challenge in rowing performance, a great part of the literature focused on these intra-cycle velocity variations; therefore, the inter-cycle velocity variations received less attention. However, several authors emphasized that inter-cycle velocity variations must be minimized in rowing (Martin and Bernfield, 1980; Baudouin and Hawkins, 2002, 2004; Soper and Hume, 2004; Nolte, 2011). The first law of Newton (law of inertia) mentions that an object will continue in a state of rest or of uniform motion (i.e., constant velocity) unless acted upon by external forces that are not in equilibrium (for reviews, see Hay, 1993; Bartlett, 2007). In cyclical locomotor activities such as rowing and swimming, fluid dynamic forces act in a direction opposite to the object's motion and are called drag forces. Drag forces resist motion and, therefore, limit speed, thus sports performance in rowing. To maintain a boat in motion at a constant speed, propulsive forces that equal the total drag force, but in opposite direction, have to be exerted. Thus, propulsive forces have a power that equal the product of the drag force and the speed. From there, the aim of rowers is to maintain a constant speed by minimizing both intra- and inter-cycle velocity variations, in order to minimize too high expenditure of energy. In rowing the minimization of inter-cycle velocity variations can be achieved in three distinct ways: (i) monitoring stroke rate (Soper and Hume, 2004 advised 30 cycle.min^−1^ for 2000 m; Hofmijster et al., 2007 advised a stroke rate considerably lower than 36 cycle.min^−1^), (ii) optimizing the ratio between stroke length and stroke rate, and (iii) increasing the synchronization between the rowers. For this latter point, Baudouin and Hawkins (2002) mentioned that “*coordination and synchrony between rowers in a multiple rower shell affects overall system velocity*” (p. 401); to improve this factor, they advised to examine how force-time profiles between rowers match, which helps to generate a balanced cumulative blade force. This coordinative aspect of rowing performance was recently investigated through the analysis of how rowers experienced their activity (Lund et al., 2012; Millar et al., 2013; R'Kiouak et al., 2016): the authors emphasized that the rowers not only attempted to coordinate their limbs (i.e., intrapersonal coordination) and themselves (i.e., interpersonal coordination), but also sought to coordinate with other environmental information such as the variations in the boat velocity (i.e., extrapersonal coordination) (Millar et al., 2013).

Managing interpersonal coordination therefore seemed more complex than just the bow rower adjusting to the stroke rower. A case study of a coxless pair crew, which combined the analysis of the phenomenological data (e.g., concerns) from stroke and bow rowers as they performed and the biomechanical characteristics of their movements, demonstrated that the rowers needed to continually adjust their interpersonal coordination (Sève et al., 2013). In particular, the biomechanical parameters studied in relation to the interpersonal coordination helped elucidate the stroke rower's perception of “being pushed.” The authors showed that the stroke rower had a bigger stroke amplitude, which involved moving more quickly during the recovery phase in order to catch up to the bow rower's movement and be synchronized for the catch (Sève et al., 2013). A second phenomenon concerning the recovery angular velocity was also evoked to explain the stroke rower's perception of “being pushed.” The stroke rower exhibited a lower angular velocity during the first part of the recovery, which led him/her to generate higher velocity during the second part of the recovery (Sève et al., 2013). These authors analyzed the crew phenomenology through their *pre-reflective self-consciousness* embedded in the lived experience, i.e. the immediate meaning that emerges from the individual's action at each instant and in which the following action is anchored (Merleau-Ponty, 1945; Varela et al., 1991). The crew phenomenology analysis was done on the basis of the lived experience, which concerned the perceptions, concerns and actions of the rowers, collected by retrospective phenomenological interviews (according to the course-of-action methodology; Theureau, 2003; Araujo and Bourbousson, 2016). The combination of phenomenological and mechanical data shed light on how the participants subjectively experienced some of the features of interpersonal coordination. As exemplified recently, it also suggested the interest of investigating how rowers in a cooperative context are able to systematically remain aware of what may perturb performance and the interpersonal coordination they are engaged in, especially when the situation is not controlled in a lab but in a race against the clock (Seifert et al., 2016a). Taken together, behavioral and phenomenological data have highlighted how individuals behave, interact and live experience within their environment (including other individuals), thereby enriching our understanding of interpersonal coordination. This type of phenomenological investigation has shed light on interpersonal coordination as being dynamically regulated (De Jaegher and Di Paolo, 2007; Froese and Di Paolo, 2011; Froese, 2012), especially in observational studies in ecological performance contexts with no constraints controlled by the experimenters.

Although a leader–follower relationship could be expected between the stroke and bow rowers, the previous studies exemplified how the interpersonal coordination was influenced by interacting constraints like weather, wind, waves, change in the river pathway, fatigue, race strategy, and partner activity (for an extensive rationale for the constraint-led approach, see Newell, 1986). Therefore, in our rowing study in a cooperative performance context, we explored crew phenomenology to determine how interacting constraints were meaningful to the rowers; that is, whether these constraints were perturbing or contributed to shaping the interpersonal coordination dynamics. In particular, we assumed that examining both the phenomenology and the dynamics of a coupled oscillator system in a coxless pair crew would provide insight into the inter-cycle variability of interpersonal coordination in an ecological context of performance.

Previous studies have already shown that movement and coordination pattern variability may have a functional and adaptive role (Newell et al., 2005; Davids et al., 2006; Seifert et al., 2014, 2016b), highlighting property of “degeneracy” (Edelman and Gally, 2001) or “functional equivalence” (Kelso, 2012) in neurobiological systems. Edelman and Gally (2001) defined degeneracy as the capacity of system components that differ in structure to achieve the same function or performance output. From this perspective, the functional characteristics of variability reflect the adaptability to reach a task-goal and maintain a high level of performance. Adaptive behaviors, in which system degeneracy is exploited, occur when perceptual motor system is stable when needed and flexible when relevant (Warren, 2006; Seifert et al., 2016b). Thus, although neurobiological systems naturally tend to remain relatively stable within a specific context for reasons of energy efficiency and economy (Sparrow and Newell, 1998) stability and flexibility are not opposite. In particular, flexibility is not a loss of stability but, conversely, is a sign of perceptual and motor adaptability to interacting constraints, in order to facilitate (structural or not) changes in coordination patterns, at the same time, maintaining functional performance (Seifert et al., 2016b). A crucial question in rowing is to understand which part of rowers' coordination is changed when a coxless pair crew adapts to interacting constraints. On one hand, stability of the rowers' coordination could mean that the coordination pattern is reproducible and consistent over time and resists perturbations (e.g., wind and waves in rowing). On the other hand, a flexible behavior means that coordination pattern is not stereotyped and rigid, but adapts to a modification in the set of constraints (e.g., when rowers approach a turn in the river or when rowers are exhausted). This in fact illustrates how the perceptual and motor system might exploit degeneracy property.

What makes this study original is that most studies in rowing highlight the necessity of minimizing inter-cycle velocity variations, but fail to examine the relationships between inter-cycle velocity variations and the movement coordination variability of the rowers. Interestingly, this approach has been proposed in swimming, another cyclic aquatic activity. Cycle-to-cycle analysis (during three sets of 300 m swam at 70, 80, and 90% of the personal best time of the 400 m) showed that well-trained swimmers exhibited higher swimming velocity, lower inter-cycle velocity variations and higher adaptability of inter-arm coordination than recreational swimmers (Dadashi et al., 2016). These authors concluded “*movement pattern variability showed that skilled swimmers could faster adapt to a new task-environmental constraint, suggesting that cycle velocity variation can be used as a prevalent metric to distinguish the technical capacity of swimmers*” (p. 8) (Dadashi et al., 2016). This exemplifies Seifert et al. (2014, 2016b) conclusion, that property of degeneracy in perceptual and motor systems supports functional movement coordination variability. Based on the similarities existing between swimming and rowing, (i.e., cyclical skills taking place in an aquatic environment with alternation of underwater propulsion and aerial recovery), previous studies on swimmers suggest that variability in motor coordination can be considered as functional when (i) velocity of locomotion is high and (ii) is associated with low inter-cycle variations. In such case, this variability reflects the degeneracy of the perceptual and motor systems to adapt to the set of constraints.

The first aim of this study was to examine the variability of the interpersonal coordination and of the individual organization in relation to rowing performance, to better understand the leader-follower relationships. Indeed, the analysis of rower movement variability and of interpersonal coordination variability might inform on how rowers exploit perceptual and motor systems degeneracy. To reach this aim, we conducted a descriptive and exploratory study of two coxless pair crews performing a 3000-m race against the clock without manipulating any constraints. The second aim was to analyze the crew phenomenology in order to understand how the rowers experienced their own action and their joint action when they had to cope with naturally occurring race constraints. As our investigation was conducted in an ecological context of performance, we postulated that combining the data on crew phenomenology with our analysis of the behavioral dynamics of interpersonal coordination and individual organization would enrich our understanding of the role of variability (for more details, see Seifert et al., 2016a) and degeneracy property. Depending on how the performance evolved (decrease vs. maintenance of high average boat velocity), we hypothesized that the race constraints would lead to perturbations or functional adaptations in the interpersonal coordination and/or individual organization, which would be experienced by the two rowers (a) simultaneously or not simultaneously, (b) as meaningful or meaningless, and (c) as similar or diverging concerns.

## Materials and methods

### Participants and protocol

This study presents two case studies; therefore, it is difficult to generalize the results and to run any statistical analysis. Two coxless pair crews participated in this study: an international men's pair (lightweight) and a national women's pair (junior). The characteristics of the stroke rower of the international crew were: age 26 years, height 187 cm and weight 67 kg; he had 12 years of rowing experience and trained 20 h/week. He was the national champion twice (2009–2010), won the World Cup in 2008, and ranked fourth at the 2008 Olympics Games. The characteristics of the bow rower were: age 30 years, height 183 cm and weight 70 kg; he had 16 years of rowing experience and trained 20 h/week. He was the national champion in 2009 and ranked second at the national championships in 2008; he ranked fourth in the World Cup in 2008 and fourth at the World Championships in 2009. This pair was chosen for the study primarily because both rowers had extensive experience and expertise in rowing and had been rowing together at the top level for 4 years. Conversely, the women of the national junior crew had never rowed together in competition and had only trained together three times. They also had less experience and expertise in rowing than the international men's pair, suggesting that they might exhibit less skill in adapting to each other. Moreover, the stroke rower was a bit more experienced than the bow rower and the coach expected an asymmetric and unbalanced relationship between them. The characteristics of the stroke rower of the national women's crew were: age 18 years, height 178 cm and weight 82 kg; she had 4 years of rowing experience and trained 15 h/week. She was ranked second at the national junior championships in 2008, fourth at the World Championships in 2008 and fifth in 2009. The characteristics of the bow rower of the national women's crew were: age 17 years, height 188 cm and weight 80 kg; she had 3 years of rowing experience and trained 15 h/week. She was ranked fifth at the World Junior Championships in 2009.

The study was designed and conducted in close collaboration with their coaches. The coxless pair is a boat for two rowers, a stroke rower and a bow rower, each having a single oar. The rowing activity was studied during a 3000-m race against the clock. The men's pair had a run of 350 oar strokes in 10′51″96 while the women's pair had a run of 373 oar strokes in 13′10″10. Both runs were performed in the same pathway on different dates. Since this experiment was performed in ecological conditions (on-water), weather conditions were not identical between crews. According to the coach's verbal report, the wind was noticeably stronger for the men's pair than for the women's pair.

This study was carried out in accordance with the recommendations set out in the guidelines of the International Committee of Medical Journal Editors. The ethics committee of Nantes University approved the protocol. The protocol was explained to all participants, who then gave written informed consent in accordance with the Declaration of Helsinki; in particular, the parents of the junior pair gave their consent.

### Mechanical measurements

Data were collected during the race using the *Powerline* system (Peach Innovations, Cambridge, UK, http://www.peachinnovations.com). This system has a data acquisition and storage center connected to (a) two sensors to measure the forces applied at the pin of each oarlock (in the direction of the longitudinal axis of the boat), (b) two sensors to measure each oar angle in the horizontal plane (angle between the oar and the axis perpendicular to the longitudinal axis of the boat), and (c) an accelerometer and a speed sensor (impeller fixed to the hull of the boat) placed at the center of the boat (for further details, see R'Kiouak et al., 2016). The accuracy of the force and angle sensors is respectively 2% of full scale (1500 N) and 0.5°, and data were sampled at 50 Hz (Coker et al., 2009). The drive phase begins with a minimum oar angle (catch) and ends with a maximum angle (finish), and conversely for the recovery phase (Hill, 2002; Sève et al., 2013).

### Phenomenological data collection

The rowers' behaviors and verbal communications (both rowers were equipped with microphones) were recorded during the entire race with two video cameras. The race was filmed from a boat that followed the coxless pairs. To capture the rowers' phenomenology through their *pre-reflective self-consciousness* embedded in the unfolding activity (i.e., lived experience) (Merleau-Ponty, 1945; Varela et al., 1991), our study included a methodology for retrospective phenomenological interviews (according to the course-of-action methodology; Theureau, 2003; Araujo and Bourbousson, 2016). Essentially, we conducted self-confrontation interviews immediately after the race to collect the phenomenological data that reflected their *pre-reflective self-consciousness* (as extensively developed in the cognitive ergonomics field; Theureau, 2003; Mollo and Falzon, 2004). This pre-reflective self-consciousness characterizes the immediate experience for individuals; that is, the *meaning* that emerges from their action at each instant “*t*” for a given period and in which the following action is anchored Merleau-Ponty, 1945; Varela et al., 1991; Theureau, 2003. The pre-reflective self-consciousness is the meaningful part of an individual's activity and situation: the individuals can *show it* (i.e., the activity can be mimed by the individual and the elements taken into account in the situation can be pointed out), *tell it* (i.e., the elements of the situation and activity that are pertinent from the individual's point of view can be described) and *comment on it* (i.e., certain elements of the activity and situation can be connected with other elements) at each instant under certain methodological conditions of confrontation (i.e., relationship of trust between rower and researcher; focusing the rower on the immediate activity with specific questioning) with the behavioral traces of their activity (Theureau, 2003). Thus, the “*meaningfulness*” of the situation reflects the individual's capacity to construct meanings during the course of his/her activity in relation to the subjective appropriation of the events encountered. Individuals interact only with the environmental elements that are sources of perturbation to the dynamics of their own activity. Therefore, the meaningfulness of the situation characterizes his/her “own world” (i.e., “Umwelt”; von Uexküll, 1992) in which the individual operates to drive the course of his activity (according to the enactive approach developed by Varela et al., 1991). In our study, video recordings collected the behavioral traces of activity during the race. The interviews were based on these video recordings and consisted of confronting each rower with his/her activity. The participants viewed these videotapes while respecting the race chronology. Immediately after each race they were invited to reconstruct and share their own lived experience, which concerned their perceptions (e.g., informational variables such as visual, kinesthetic, haptic, acoustic variables), concerns (e.g., purposes and concerns) and actions (e.g., communications between rowers, actions with the oar). In this way, the researcher was able to more fully focus on the dynamics of the individual's concerns in the situation and the dynamics of what was meaningful for the individual at each instant. Before each interview, the researcher/interviewer reminded the participant of the nature of the interview and the expectation that the participant needed to “re-live” and describe his/her own experience during the race, without any prior analysis, rationalization or justification (Theureau, 2003). This method is designed to reach the level of activity that is *meaningful* for the individual at his/her pre-reflective level of consciousness. Thus, the goal of the self-confrontation interview is to encourage the participants to verbally report what they did, felt, thought, and perceived during the race, as naturally as possible, from their own perspective (Theureau, 2003). A number of recent empirical studies in the field of sports expertise have demonstrated the fruitfulness of this methodology for studying the activity–situation coupling during interpersonal coordination tasks (Bourbousson et al., 2011, 2012; Poizat et al., 2012, 2013). Researchers who had already conducted self-confrontation interviews of this type in previous research conducted all the interviews.

### Interpersonal coordination analysis

Raw data (oar angles, forces applied to the oarlocks, acceleration and velocity) were filtered with a low pass Butterworth filter with a 5-Hz cutoff frequency. Continuous angular velocities were then computed as the first derivative of the angular position using the central difference formula. In line with de Brouwer et al. (2013) and McGarry et al. (1999), interpersonal coordination was assessed using the continuous relative phase (ϕ_rel_, in degrees) between two oscillators (i.e., oar angles of the stroke and bow rowers). In accordance with Hamill et al. (2000), the data on angular displacements (θ_norm_) and angular velocities (ω_norm_) were normalized in the interval [−1, +1] cycle to cycle. Then phase angles (ϕ_stroke_ and ϕ_bow_, in degrees) were calculated and corrected according to their quadrant (Hamill et al., 2000):

(1) $$\begin{array}{l} \phi =\text{arctan}\left(\omega_{\text{norm}}/\theta_{\text{norm}}\right) \end{array}$$

Last, the continuous relative phase for a complete cycle was calculated as the difference between the two phase angles (Hamill et al., 2000):

(2) $$\begin{array}{l} \phi_{\text{rel}}=\phi_{\text{stroke}}-\phi_{\text{bow}} \end{array}$$

Following the method of Hill (2002), the kinetic analysis of interpersonal coordination related to the *area* differences (used to estimate the applied power) and *form* differences (used to estimate the movement pattern). The area under the force–time curve differences corresponded to the force impulse differences between the rowers. The force impulse was computed for each cycle of each rower as the area under the force–time curve. Then, the force impulse differences of the two rowers were computed cycle to cycle. Second, the form differences corresponded to the force–time shape differences that we studied through continuous relative phase. The continuous relative phase was calculated from the force–time curves of the two rowers, using the previous equations detailed for kinematic analysis.

### Individual organization analysis

The oar angle–time and force at oarlock–time series of the two rowers of the same crew were compared by Student *t*-tests in order to detect which rower was responsible for the interpersonal coordination variability. Statistics were performed with Statistica 8.0 with a level of significance fixed at *p* < 0.05.

### Inter-cycle variability in interpersonal coordination and individual organization

Each cycle was considered between catch points as the local minimum of the oar angle. Then, force and angle data were resampled to 101 points per cycle, in order to make comparisons between cycles (with cycles of similar duration). The *inter-cycle variability* was assessed with the root mean square (*RMS*) and the Cauchy index (*C*_*i*_) (Chen et al., 2005; Rein, 2012). *RMS* measures the similarity between each cycle and the mean cycle of the time series, while *C*_*i*_ measures the similarity between two successive cycles of the time series. The calculation of *RMS* is based on the squared Euclidean distance between two time series at each point that is averaged, and the square root is taken:

(3) $$\begin{array}{l} RMS_{i}=\sqrt{\frac{{\sum^{\text{​}}}_{n\;=\;1}^{N}{\left(Xi_{n}-{\bar{X}}_{n}\right)}^{2}}{N}} \end{array}$$

where *N* is the number of samples per cycle (i.e., 101 in the present case) and *Xi* the cycle, with $\bar{X}$ being the average cycle. This means that the 101 data points of *Xi* were compared with the 101 data points of $\bar{X}$. Thus, a small value of *RMS* informs about similar patterns of coordination in comparison with the average pattern. *C*_*i*_ is based on the Euclidian distance that separates two successive cycles during a trial:

(4) $$\begin{array}{l} C_{i}=\frac{1}{K^{*}\left(N-1\right)}\sum_{n\;=1}^{N}\;\sqrt{\sum_{k=1}^{k}{\left(x_{kn(i\;+\;1)}-x_{kn(i)}\right)}^{2}} \end{array}$$

where *i* corresponds to a cycle, *K* the number of variables (i.e., the value of continuous relative phase or force difference in the present case), and *N* the number of samples per variable during one cycle (i.e., 101 in the present case) (Chen et al., 2005; Rein, 2012). Thus, a small value of *C*_*i*_ informs about similar successive patterns of coordination without defining the nature of the pattern. *RMS* and *C*_*i*_ were computed for the continuous relative phase between the oar angles of the bow and stroke rowers, the continuous relative phase between the oarlock forces of the bow and stroke rowers, and the force impulse differences between the rowers. For both *RMS* and *C*_*i*_, when the cycle was within the 95% confidence interval (i.e., average cycle ± 1.96 *SD*), it was considered as not perturbed.

### Inter-cycle velocity variations of the boat

The acceleration signal was integrated to provide instantaneous boat velocity variations and then to obtain the average velocity for each cycle. Because drift may occur from the acceleration signal, the average velocity obtained from the accelerometer was aligned on the average velocity calculated from the speedometer. Once the average velocity was computed for each cycle, the average boat velocity, its standard deviation and then its confidence interval were calculated, in order to determine the cycles outside of the 95% confidence interval.

### Combination of behavioral data and performance

The kinematic and kinetic parameters of behavior were then combined with the performance indicators in order to gain insight into the functional and adaptive aspects of the interpersonal coordination variability throughout the race. As emphasized in the introduction, rowers can adapt to a set of race constraints by varying their motor behaviors (structurally) without compromising function (i.e., to maintain stable boat velocity that remains within the 95% confidence interval), providing evidence for neurobiological system degeneracy (Edelman and Gally, 2001; Seifert et al., 2014, 2016b). Therefore, the property of degeneracy in perceptual and motor systems supports the functional variability of interpersonal coordination when it was associated with performance stability; that is, high average velocity (for an extensive discussion about this functional and adaptive aspect of coordination variability in relation to its impact on performance stability, see Davids et al., 2003, 2006; Seifert et al., 2014). The variability of behavior and performance was considered significantly high when the cycle was outside the 95% confidence interval. From there, three scenarios were distinguished to determine whether the behavioral variability was functional and adaptive (i.e., without significant change in boat velocity) or associated with perturbation (i.e., with significant change in boat velocity) in the coupling between rowers: (a) *functional adaptation*: at least one behavioral parameter (kinematic or kinetic) was perturbed but the boat velocity was not perturbed, (b) *behavioral perturbation*: at least one behavioral parameter (kinematic or kinetic) and the boat velocity were perturbed, and (c) *velocity perturbation*: no perturbation of the behavioral parameters but the boat velocity was perturbed.

### Analysis of the phenomenological data and their combination with behavioral data

The verbalization data from the self-confrontation interviews were processed according to the procedure defined in the course-of-action methodology (Theureau, 2003), which follows a comprehensive and idiosyncratic approach and is grounded in the enactive approach (Varela et al., 1991; Stewart et al., 2010; Araujo and Bourbousson, 2016). We therefore followed five steps:

The first step consisted of *generating a summary table containing the data recorded during the race* (i.e., a brief description of each rower's behavior) and *the self-confrontation interview* (i.e., verbatim transcriptions of the prompted verbalizations).

The second step consisted of *identifying the elementary units of meaning* (EUMs), which are the smallest units of activity that are meaningful for an individual. This process was accomplished by analyzing the audio-video recordings together with the verbalization transcripts.

The third step consisted of *reconstructing each rower's personal course of action*, leading to the identification of the *concerns* within each EUM that were meaningful to each rower. The course of action is the reduction of the phenomena of human activity to the level of “acceptable symbolic description” (Varela, 1989, p. 184) and is a valid and useful explanation of the activity. This takes into account the individual's construction of meaning for his/her activity as it unfolds and the “extrinsic” characteristics that the individual considers meaningful (Theureau, 2003). Therefore, the reconstructions of the rowers' courses of action consisted of identifying and documenting the components of the EUMs. Three inseparable components were identified and documented in this study: the *unit of course of action*, the *representamen* and the *concerns*. The *unit of course of action* is the fraction of pre-reflective activity that can be shown, told, and commented on by the individual. The unit of course of action may be a symbolic construct, physical action, interpretation, or emotion. The *representamen* corresponds to the elements that are taken into account by the individual at a given moment. The representamen may be perceptive or mnemonic. The *concerns* refer to the inherent interest of the rower's current activity based on what is meaningful to him/her. In our study, we focused particularly on the “meaningfulness” of the concerns; that is, what the rowers really took into account in the environment in order to act. Therefore, concerns were “meaningless” when the rowers could not put his/her concerns into words or when the researcher could not infer the concerns from the recordings of their behaviors and verbal communications.

The fourth step consisted of *identifying the typical concerns* of the rowers. *Typicality* refers to at least four aspects that researchers use to identify occurrence-types (Durand, 2014): (a) they concentrate the most attributes of the activity being observed in the sample of individuals and situations under study, (b) they are most frequently observed in the sample, (c) they show a propensity to occur preferentially when conditions having a “family resemblance” to those being observed are produced, and (d) the individuals express a sentiment of typicality about them in their interactions with the researchers.

The fifth step consisted of *characterizing the shared experience* of the two rowers. To do so, we analyzed each rower's personal course of action and compared them in order to understand whether the typical concerns of the two rowers led them to: (a) simultaneous or not simultaneous, (b) meaningful or meaningless, and (c) similar or diverging concerns. These three criteria were used to characterize the rowers' shared experiences in four collective phenomenological categories (for a similar study, see (R'Kiouak et al., 2016)). The first collective phenomenological category was labeled Simultaneously and Similarly Experienced as Meaningless (SSE-L) when the rowers did not pay attention to the joint action at the pre-reflective level of their activity. The second category was labeled Simultaneously and Similarly Experienced as Meaningful (SSE-F) when the rowers reported a salient, meaningful experience of the joint action to cope with the race constraints. The third category was labeled Simultaneously Diverging Experiences (SDE) when the joint action was associated with diverging concerns (i.e., not similarly experienced). The fourth category was labeled Not Simultaneously Experienced as Meaningful (NSEM) when one rower reported a meaningful experience of the joint action whereas the other rower did not pay attention to it. Table 1 shows examples of the concerns of the stroke and bow rowers of the international crew, analyzed to determine their simultaneity, meaning and convergence and categorized into one of the four collective phenomenological categories.

**Table 1:** **Examples of concerns of the international crew stroke and bow rowers, analyzed to determine the simultaneity, meaning and divergence of these concerns between rowers and assigned to one of the four collective phenomenological categories**.

**Time (s)**	**Perturbing vs. functional variability of behavior and performance**	**Concerns of the stroke rower**	**Concerns of the bow rower**	**Similarity or divergence of concerns between rowers**	**Shared experience**
62.3–66.0	Functional adaptation	x	Control the direction to turn the boat	Divergence because this functional adaptation was meaningless for the stroke rower and the bow rower wanted to turn the boat	NSEM
274.8	Functional adaptation	x	Focus on his technique (catch phase)	Divergence because this functional adaptation was meaningless for the stroke rower and the bow rower focused on his technique	NSEM
406.0–413.7	Behavioral perturbation	Try to come back to a comfortable situation after the wave, try to keep the pace	Be synchronized with the stroke rower	Divergence because the stroke rower focused on the boat and wave whereas the bow rower focused on his partner	SDE
539.8	Behavioral perturbation	Remain lucid, focused on technique till the end; be vigilant about information provided by the bow rower about the waves; anticipate waves	Focus on his partner; request the stroke rower to keep the boat up to river level	Divergence because the stroke rower focused on his technique whereas the bow rower focused on his partner	SDE
623.4	Functional adaptation	Focus on the final part; save time	Initiate the final part progressively by being synchronized with his partner	Divergence because the stroke rower focused on his stroke frequency and boat velocity whereas the bow rower focused on his partner	SDE

*The last column indicates whether the stroke and bow rowers experienced this higher variability in joint action and/or performance as (a) Simultaneously and Similarly Experienced as Meaningless (SSE-L), (b) Simultaneously and Similarly Experienced as Meaningful (SSE-F), (c) Simultaneous and Diverging Experiences (SDE), or Not Simultaneously Experienced as Meaningful (NSEM), on the basis of the phenomenological data*.

Last, the sixth step consisted of *combining the phenomenological data with the behavioral and performance data* to determine whether the functional adaptations and behavioral perturbations were associated with (a) simultaneous or not simultaneous, (b) meaningful or meaningless, and (c) similar or diverging concerns of the two rowers.

Several measures were taken to enhance the validity of this analysis (Lincoln and Guba, 1985). First, the self-confrontation interviews were conducted in an atmosphere of trust between rowers and researchers. Trust was built via the establishment of an explicit contract between the researcher and the participant that took into account the respective interests of each one. Second, two investigators independently carried out the data analysis (i.e., reconstructing the courses of action and identifying the typical concerns, then how these concerns were shared by the rowers) and discussed any initial disagreement until a consensus was reached. These two researchers had already coded protocols of this type in previous studies and were accustomed to course-of-action methodology. This method is justified by the particular characteristics of data analysis in this methodology. Indeed, reconstructing a course of action is not strictly a coding procedure: it requires a plausible interpretation of the ongoing construction of meaning during the individual's activity. This is ensured by the parallel data analysis by different researchers, who mutually discuss their interpretations. Third, a saturation criterion was adopted for the categorization of *typical concerns*. This criterion was considered to be met when no new categories of *typical concerns* emerged from the processing of further data.

## Results

**Figure 1:**
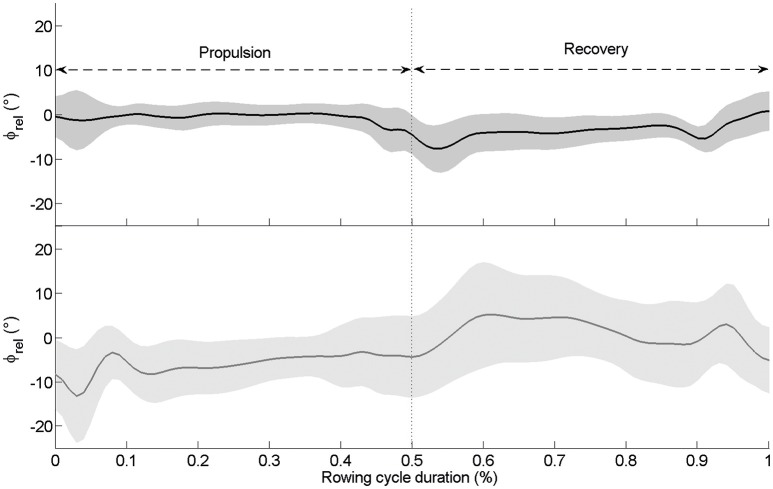
**Mean continuous relative phase (ϕ_**rel**_) for kinematic data calculated on the total number of cycles for the international crew (top panel: black line)** and the national crew **(low panel: gray line)**. The gray zone around the ϕ_rel_ curve represents the standard deviation. The propulsion goes from 0 to 0.5 (~50% of the cycle duration) while the recovery goes from 0.5 to 1.

**Figure 2:**
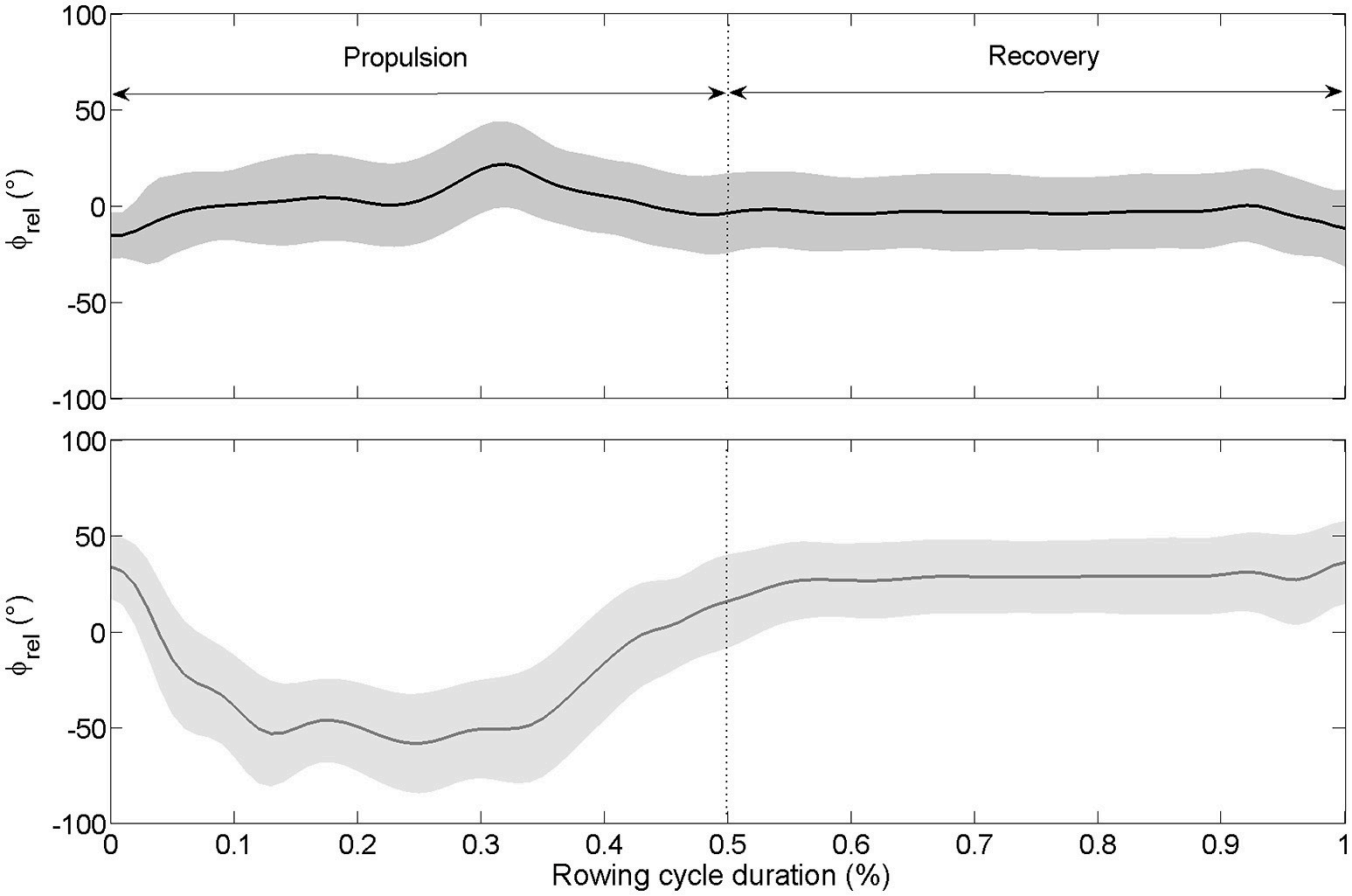
**Mean continuous relative phase for kinetic data (ϕ_**rel**_ between oarlock force–time shape) calculated on the total number of cycles for the international crew (top panel)** and the national crew **(low panel)**. The gray zone around the ϕ_rel_ curve represents the standard deviation. The propulsion goes from 0 to 0.5 (50% of the cycle duration) while the recovery goes from 0.5 to 1.

The oar angle–time curves (Figure 1) and the force at oarlock–time curves (Figure 2) of the bow and stroke rowers showed in-phase coupling between rowers. However, when the interpersonal coordination was computed for the continuous relative phase from the oar angles, continuous relative phase from the oarlock forces and force impulse difference, variability was noted between cycles.

### Inter-cycle variability in interpersonal coordination

**Figure 3:**
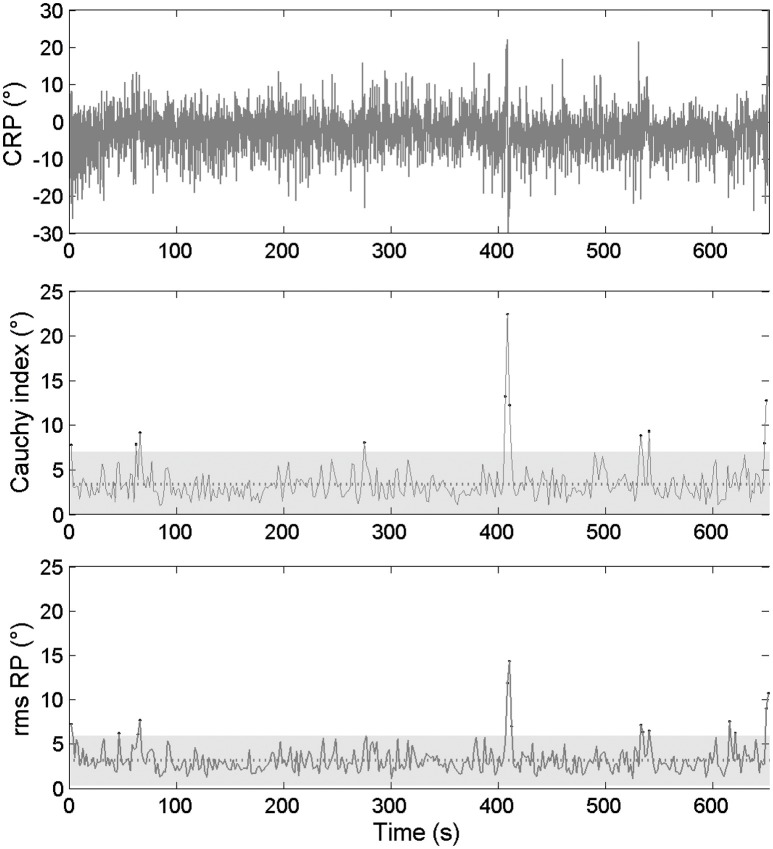
**Continuous relative phase time series for kinematic data and the related RMS and ***C***_***i***_ time series for the international crew. (Top panel)** Represents the ϕ_rel_ between oar angles. **(Middle panel)** Represents the *C*_*i*_ calculated on ϕ_rel_ from cycle i to i+1 as its mean value and confidence interval. **(Lower panel)** Represents the RMS calculated on ϕ_rel_. Dots stand for moments when *C*_*i*_ and RMS values are outside of their confidence intervals (gray zone).

**Figure 4:**
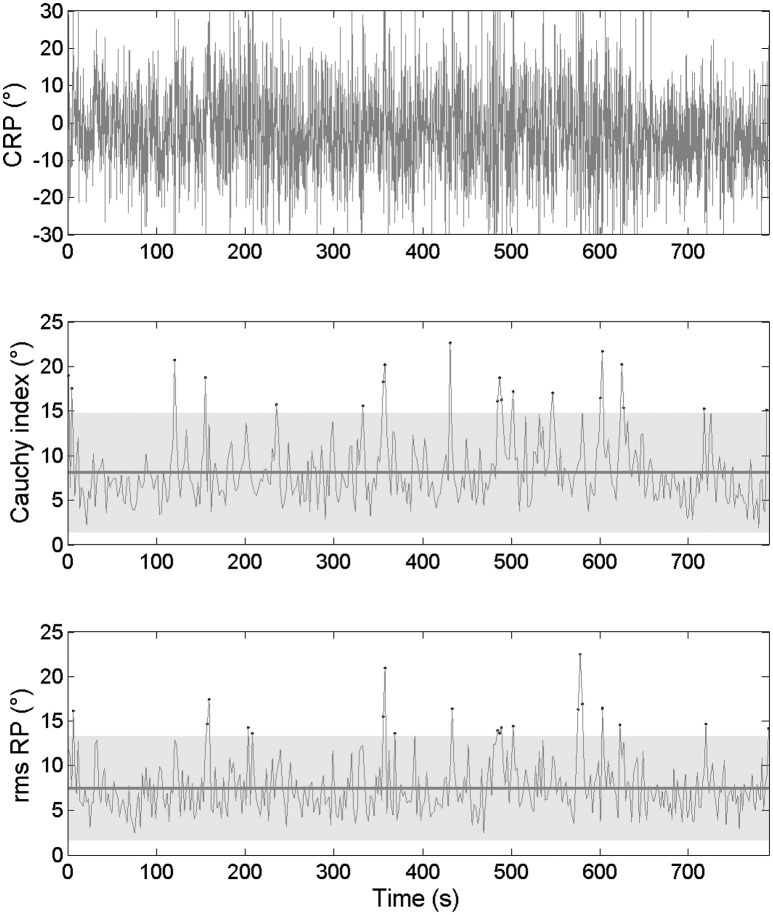
**Continuous relative phase time series for kinematic data and the related RMS and ***C***_***i***_ time series for the national crew. (Top panel)** Represents the ϕ_rel_ between oar angles. **(Middle panel)** Represents the *C*_*i*_ calculated on ϕ_rel_ from cycle i to i+1 as its mean value and confidence interval. **(Lower panel)** Represents the RMS calculated on ϕ_rel_. Dots stand for moments when *C*_*i*_ and RMS values are outside of their confidence intervals (gray zone).

The inter-cycle variability was examined through its *magnitude* (*RMS* and *C*_*i*_ values) and *frequency* (number of cycles outside of the confidence interval, based on *RMS* and *C*_*i*_ data). Concerning the kinematic data, the international crew exhibited a mean *RMS* ϕ_rel_ = 3.21 ± 1.42, with 11 cycles outside of the confidence interval and a mean *C*_*i*_ ϕ_rel_ = 3.42 ± 1.97, with 8 cycles outside of the confidence interval for 340 cycles performed during the race (Figure 3). The national crew showed a mean *RMS* ϕ_rel_ = 7.53 ± 2.99, with 18 cycles outside of the confidence interval and a mean *C*_*i*_ ϕ_rel_ = 8.13 ± 3.50, with 17 cycles outside of the confidence interval for 363 cycles performed during the race (Figure 4).

**Figure 5:**
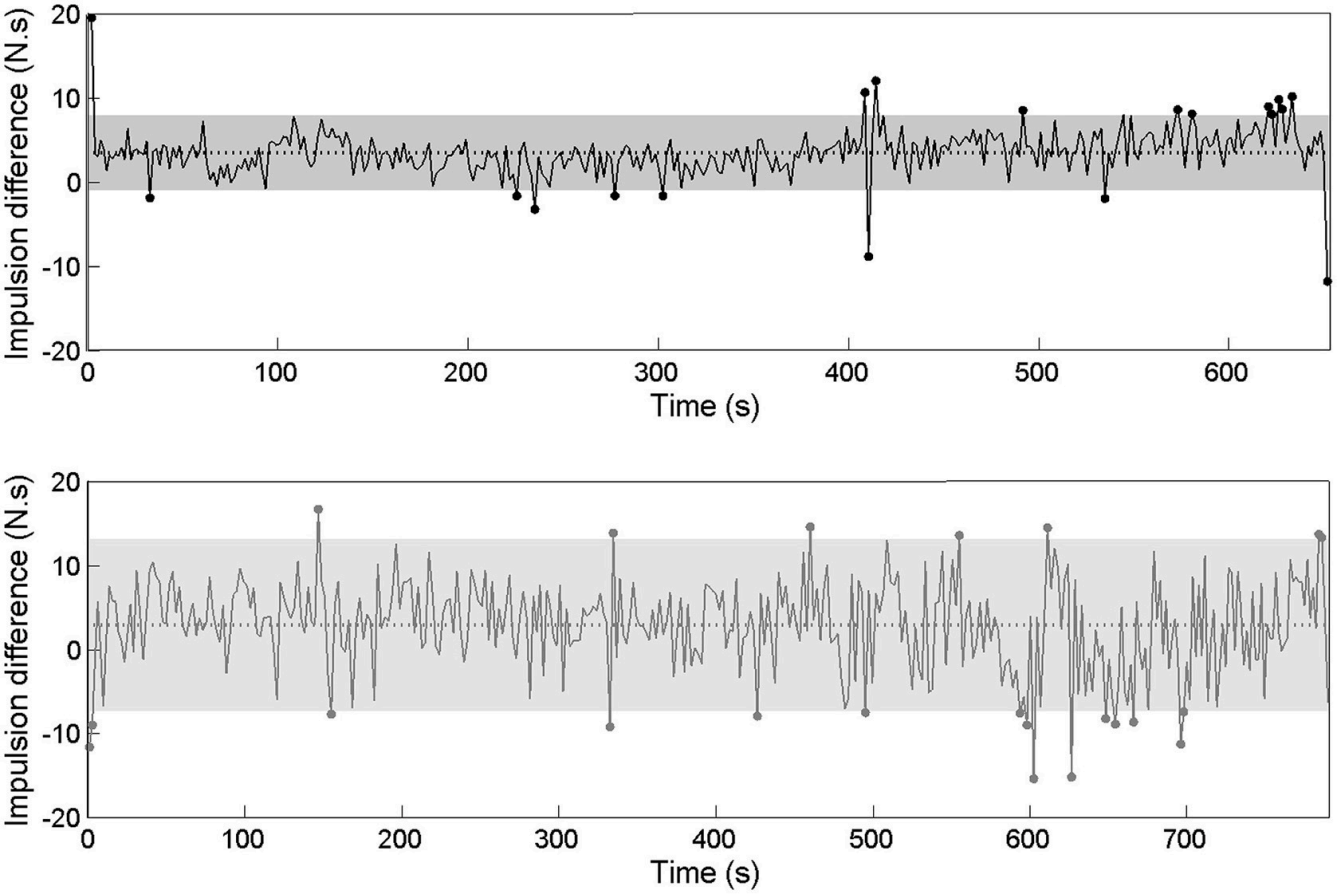
**Force impulse difference time series for the international crew (top panel: black line)** and the national level crew **(low panel: gray line)**. The gray zone represents a 95% confidence interval (1.96 *SD*).

Concerning the kinetic analysis, the international crew showed a mean force impulse difference between rowers of 3.65 ± 2.19 N.s with 17 cycles outside of the confidence interval, while the national crew exhibited a mean force impulse difference of 4.93 ± 3.38 N.s with 18 cycles outside of the confidence interval (Figure 5).

**Figure 6:**
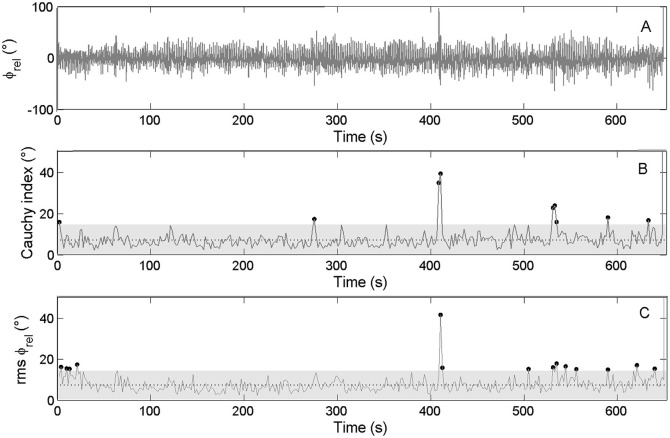
**Continuous relative phase time series for kinetic data (ϕ_**rel**_ between oarlock force–time shape) and the related RMS and ***C***_***i***_ time series for the international crew. (Top panel)** Represents the ϕ_rel_ between oarlock forces. **(Middle panel)** Represents the *C*_*i*_ calculated on ϕ_rel_ from cycle i to i+1 as its mean value and confidence interval. **(Lower panel)** Represents the RMS calculated on ϕ_rel_. Dots stand for moments when *C*_*i*_ and RMS values are outside of their confidence intervals (gray zone).

**Figure 7:**
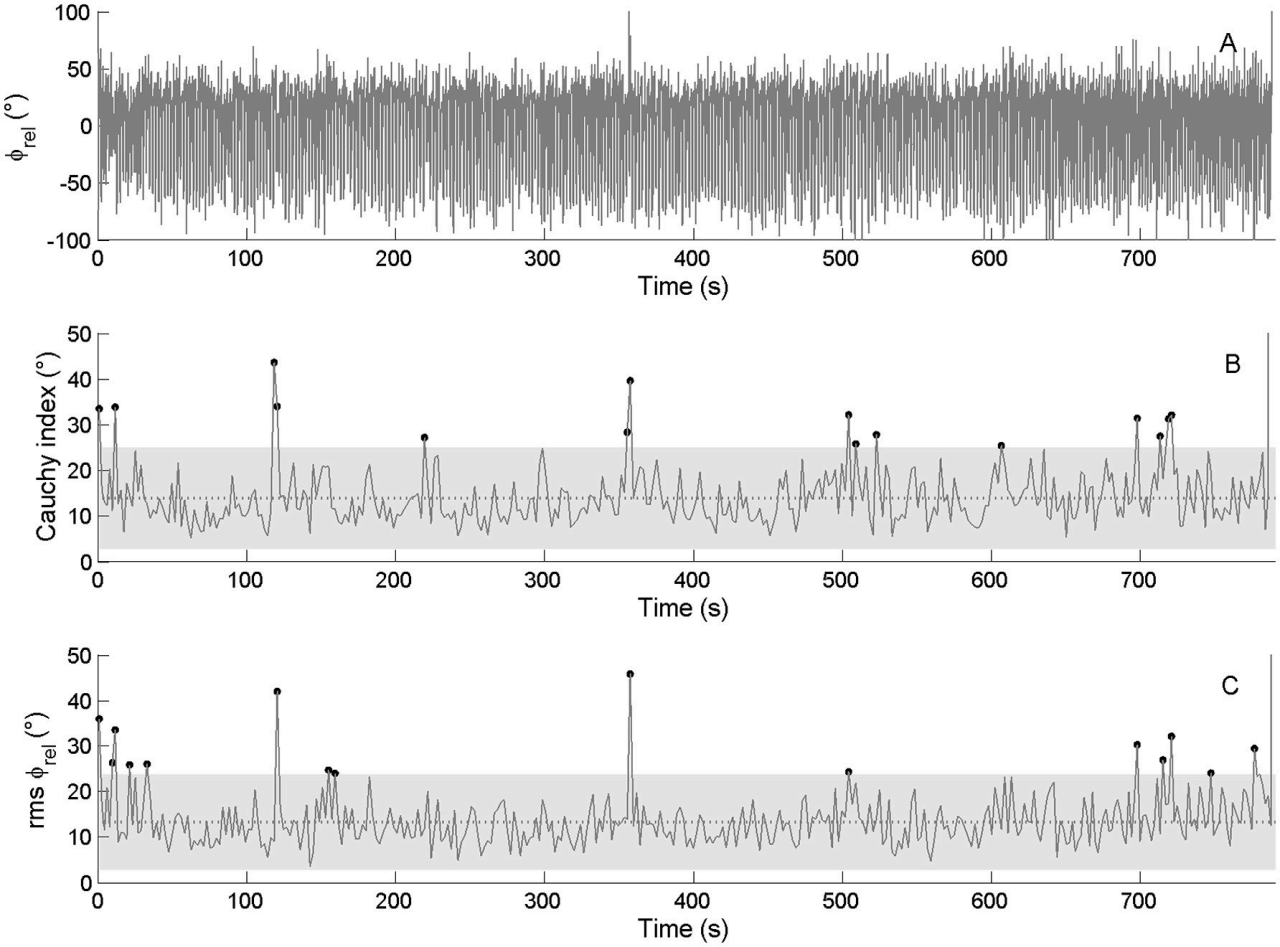
**Continuous relative phase time series for kinetic data (ϕ_**rel**_ between oarlock force–time shape) and the related RMS and ***C***_***i***_ time series for the national crew. (Top panel)** Represents the ϕ_rel_ between oarlock forces. **(Middle panel)** Represents the *C*_*i*_ calculated on ϕ_rel_ from cycle i to i+1 as its mean value and confidence interval. **(Lower panel)** Represents the RMS calculated on ϕ_rel_. Dots stand for moments when *C*_*i*_ and RMS values are outside of their confidence intervals (gray zone).

The calculation of *RMS* and *C*_*i*_ for the ϕ_rel_ on the kinetic data showed a mean *RMS* ϕ_rel_ = 7.6 ± 3.5, with 11 cycles outside of the confidence interval for the international crew, and a mean *C*_*i*_ ϕ_rel_ = 7.2 ± 3.9, with 8 cycles outside of the confidence interval for 340 cycles performed during the race (Figure 6). For the national crew, the mean *RMS* ϕ_rel_ = 13.5 ± 5.3, with 14 cycles outside of the confidence interval, and the mean *C*_*i*_ ϕ_rel_ = 13.9 ± 5.7, with 14 cycles outside of the confidence interval for 363 cycles performed during the race (Figure 7).

### Inter-cycle variability in individual organization

**Figure 8:**
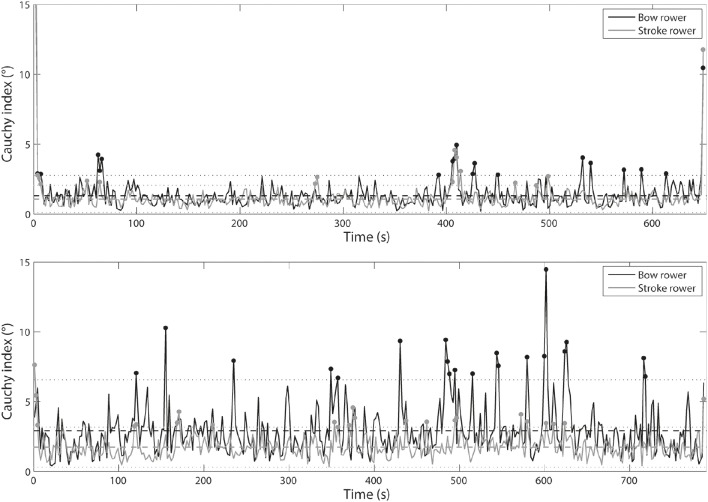
**Comparison of ***C***_***i***_ time series between stroke and bow rowers for international crew (top panel)** and national crew **(lower panel)** concerning oar angle.

Figure 8 shows individual *C*_*i*_ based on the oar angles. Whatever the crew, statistical analysis showed higher *C*_*i*_ values for the bow rower (*t* = 2.39, *p* = 0.017 for the international crew and *t* = 10.84, *p* < 0.001 for the national crew) than for the stroke rower. The stroke rower from the international crew exhibited 13 cycles outside of the confidence interval, whereas the bow rower exhibited 17 cycles outside of it, with the stroke and bow rowers both outside of the confidence interval for 9 of these cycles. In the national crew, the stroke rower exhibited 22 cycles outside of the confidence interval, whereas the bow rower exhibited 21 cycles outside of it, with both rowers together outside of the confidence interval for 8 of these cycles.

**Figure 9:**
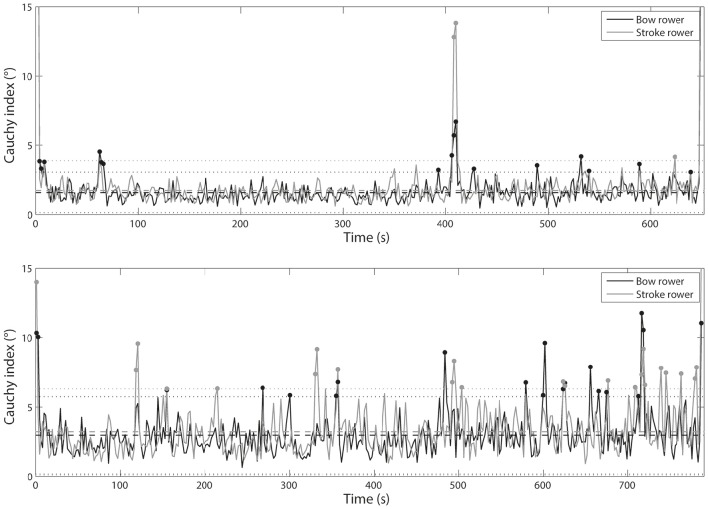
**Comparison of ***C***_***i***_ time series between stroke and bow rowers for international crew (top panel)** and national crew **(lower panel)** concerning oarlock force.

Figure 9 shows individual *C*_*i*_ based on the oarlock force production. No significant *C*_*i*_ differences were noted between the two rowers of the international crew (*t* = −1.07, *p* = 0.287) although significant differences occurred for the national crew (*t* = −2.20, *p* = 0.028). The stroke rower from the international crew exhibited 4 cycles outside of the confidence interval, while the bow rower exhibited 16 cycles outside of it, with the two rowers together outside of the confidence interval for 3 of these cycles. In the national crew, the stroke rower exhibited 21 cycles outside of the confidence interval and the bow rower exhibited 20 cycles outside of it, with the two rowers together outside of the confidence interval for 8 of these cycles.

### Inter-cycle velocity variations in the boat

**Figure 10:**
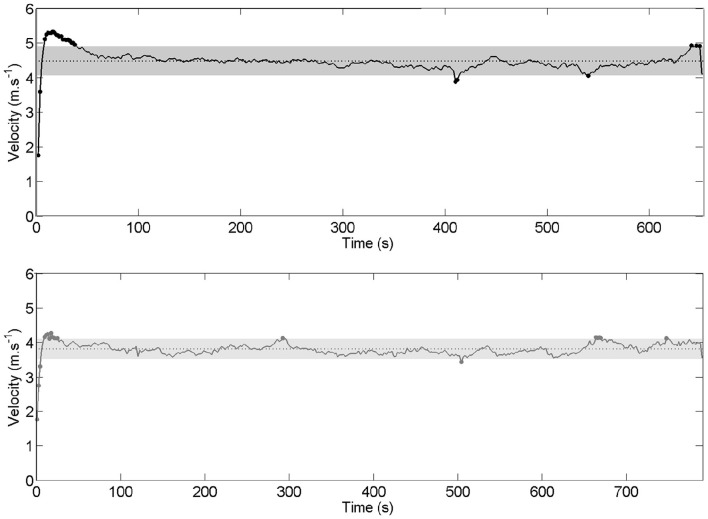
**Instantaneous velocity and average velocity over the whole race for international crew (top panel:** black line) and national crew **(lower panel**: gray line**)**. Gray zone represents the 95% confidence interval (1.96 *SD*).

The average boat velocity was 4.47 ± 0.27 m.s^−1^ for the international crew and 3.80 ± 0.19 m.s^−1^ for the national crew. Twenty-five cycles (distributed over 4 sequences) for the international crew and 21 cycles (distributed over 5 sequences) for the national crew were outside of the confidence interval (Figure 10).

### Combination of behavioral data and performance outcome

When performance (boat velocity) and the behavioral parameters (i.e., kinematic and kinetic) were combined, 16 cycles were identified as outside of the confidence interval for the international crew and could be categorized as follows: 12 cycles (75% out of a total of 16 cycles) corresponded to *functional adaptation*, whereas 4 cycles (25%) corresponded to *behavioral perturbation* (Table 2).

**Table 2:** **Number of cycles outside of the confidence interval and the time at which this high variability occurs during the race, based on boat velocity and the behavioral data (kinematic and kinetic) for the international crew**.

**Cycle number**	**Kinematic coordination**	**Kinetic coordination**	**Impulsion differences**		**Mean velocity**	**Consequences for interpersonal coordination**	**Experience of joint action and/or performance outcome**			
			**Time**	**Who's higher?**			**Stroke rower experience**	**Bow rower experience**	**Shared experience**	**Similarity or divergence of concerns between rowers**
1	–	–	32.6	Bow	9.8–39.4	Behavioral perturbation	Meaningful	Meaningful	SDE	Diverging because the stroke rower wanted to go straight and the bow rower wanted to turn the boat to stay far from a buoy
2	62.3–66	–	–	–	–	Functional adaptation	Meaningless	Meaningful	NSEM	The bow rower focused on turning the boat
3	–	–	225.2	Bow	–		Meaningful	Meaningful	SSE-F	Similar because both rowers focused on the same direction (i.e., turning because they are too close the river bank)
4	–	–	234.7	Bow	–		Meaningful	Meaningful	SSE-F	Similar because both rowers focused on the same direction (i.e., to go straight)
5	274.8	274.7	276.7	Bow	–		Meaningless	Meaningful	NSEM	The bow rower focused on his technique
6	–	–	301.9	Bow	–		Meaningful	Meaningful	SSE-F	Similar because both rowers focused on the same direction (i.e., to go straight)
7	406–409.9	407.9–409.8	407.9–413.7	Stroke - Bow - Stroke	409.8–411.8	Behavioral perturbation	Meaningful	Meaningful	SDE	Diverging because the stroke rower focused on the boat and wave whereas the bow rower focused on his partner
8	–	–	490.9	Stroke	–	Functional adaptation	Meaningless	Meaningless	SSE-L	x
9	532.1	530.1–533.9	533.9	Bow	–		Meaningless	Meaningless	SSE-L	x
10	539.8	–	–	–	537.8–539.7	Behavioral perturbation	Meaningful	Meaningful	SDE	Diverging because one rower focused on his technique whereas the other focused on his partner
11	–	–	572.1	Stroke	–	Functional adaptation	Meaningless	Meaningless	SSE-L	x
12	–	–	579.6	Stroke	–		Meaningless	Meaningless	SSE-L	x
13	–	588.9	–	–	–		Meaningless	Meaningless	SSE-L	x
14	–	–	619.9–626.9	Stroke	–		Meaningful	Meaningful	SDE	Diverging because the stroke rower focused on his stroke frequency and boat velocity whereas the bow rower focused on his partner
15	–	632.2	632.2	Stroke	–		Meaningful	Meaningful	SSE-F	Similar because both rowers focused on their technique
16	–	648.7	–	–	640.6–642.3	Behavioral perturbation	Meaningful	Meaningful	SDE	Diverging because the stroke rower increased speed and stroke frequency for the final part, while the bow rower wanted to do it progressively

*The last column indicates whether the stroke and bow rowers experienced this higher variability in joint action and/or performance as (a) Simultaneously and Similarly Experienced as Meaningless (SSE-L), (b) Simultaneously and Similarly Experienced as Meaningful (SSE-F), (c) Simultaneous and Diverging Experiences (SDE), or Not Simultaneously Experienced as Meaningful (NSEM), on the basis of the phenomenological data*.

Concerning the national crew, 26 cycles were identified as outside of the confidence interval and could be categorized as follows: 21 cycles (80.8% out of a total of 26 cycles) corresponded to *functional adaptation*, whereas 3 cycles (11.5%) corresponded to *behavioral perturbation* and 2 cycles (7.7%) related to *velocity perturbation* (Table 3).

**Table 3:** **Number of cycles outside of the confidence interval and the time at which this higher variability occurs during the race, based on the boat velocity and the behavioral data (kinematic and kinetic) for the national crew**.

**Cycle number**	**Kinematic coordination**	**Kinetic coordination**	**Impulsion differences**		**Mean velocity**	**Consequences for interpersonal coordination**	**Experience of joint action and/or performance outcome**			
			**Time**	**Who's higher?**			**Stroke rower experience**	**Bow rower experience**	**Shared experience**	**Similarity or divergence of concerns between rowers**
1	–	11.7	–	–	9.9–25.2	Behavioral perturbation	Meaningful	Meaningful	SDE	Diverging because the stroke rower felt pushed by the bow rower while the bow rower focused on the boat direction
2	120.4	118.3–120.4	–	–	–	Functional adaptation	Meaningful	Meaningless	NSEM	The stroke rower felt pushed by the bow rower
3	–	–	146.6	Stroke	–		Meaningful	Meaningful	SDE	Diverging because the stroke rower focused on the boat direction while the bow rower focused on her technique
4	154.9	–	154.9	Bow	–		Meaningless	Meaningful	NSEM	The bow rower focused on her technique
5	–	219.2	–	–	–		Meaningful	Meaningful	SSE-F	Similar because both rowers focused on their technique
6	234.9	–	–	–	–		Meaningless	Meaningless	SSE-L	x
7	–	–	–	–	285.6–296.2	Velocity perturbation	Meaningful	Meaningful	SDE	Diverging because the stroke bower focused on her stroke frequency while the bow rower attempted to follow the stroke rower's stroke frequency
8	332.3	–	332.3–334.4	Bow then Stroke	–	Functional adaptation	Meaningful	Meaningful	SSE-F	Similar because both rowers focused on their technique
9	355.2–357.2	355.2–357.2	–	–	–		Meaningful	Meaningful	SSE-F	Similar because both rowers focused on the boat direction
10	430.4	–	426	Bow	–		Meaningless	Meaningless	SSE-L	x
11	–	–	459.6	Stroke	–		Meaningless	Meaningless	SSE-L	x
12	483.9–488.2	–	494.7	Bow	–		Meaningful	Meaningless	NSEM	The stroke rower focused on her technique
13	501.4	503.7–508.5	–	–	503.7	Behavioral perturbation	Meaningful	Meaningful	SDE	Diverging because the stroke rower gave instructions to the bow rower and tried to counteract her actions, which she felt were inappropriate
14	–	522.3	–	–	–	Functional adaptation	Meaningful	Meaningful	SSE-F	Similar because both rowers focused on stroke frequency
15	545.9	–	–	–	–		Meaningful	Meaningful	SSE-F	Similar because both rowers focused on the boat direction
16	–	–	554.5	Stroke	–		Meaningful	Meaningful	SDE	Diverging because the stroke rower focused on the crew while the bow rower focused on herself
17	599.7–601.8	–	593.1–601.8	Bow	–		Meaningful	Meaningless	NSEM	The stroke rower focused on her technique
18	–	606.1	610.6	Stroke	–		Meaningful	Meaningless	NSEM	The stroke rower focused on her technique
19	623.7–625.8	–	625.8	Bow	–		Meaningful	Meaningful	SSE-F	Similar because both rowers focussed on the boat direction
20	–	–	647.6	Bow	–		Meaningful	Meaningless	NSEM	The stroke rower focused on her technique
21	–	–	653.7	Bow	–		Meaningful	Meaningless	NSEM	The stroke rower focused on her technique
22	–	–	665.3	Bow	663.5–674.6	Behavioral perturbation	Meaningful	Meaningful	SDE	Diverging because the stroke rower felt pushed by the bow rower while the bow rower focused on her technique; therefore the stroke rower turned back to get information from the bow rower
23	–	697.2	695.3–697.2	Bow	–	Functional adaptation	Meaningful	Meaningful	SDE	Diverging because the stroke rower focused on the boat direction while the bow rower focused on her technique
24	–	712.5	–	–	–		Meaningless	Meaningful	NSEM	The bow rower encouraged her partner and focused on the boat speed
25	716.4	718.3–720.2	–	–	–		Meaningful	Meaningful	SSE-F	Similar because both rowers focused on technique
26	–	–	–	–	746.6–750.4	Velocity perturbation	Meaningful	Meaningful	SDE	Diverging because the stroke rower focused on her partner while the bow rower focused on herself

*The last column indicates whether the stroke and bow rowers experienced this higher variability in joint action and/or performance as (a) Simultaneously and Similarly Experienced as Meaningless (SSE-L), (b) Simultaneously and Similarly Experienced as Meaningful (SSE-F), (c) Simultaneous and Diverging Experiences (SDE), or Not Simultaneously Experienced as Meaningful (NSEM), on the basis of the phenomenological data*.

### Combination of behavioral and phenomenological data

Our first finding indicated that the *behavioral and velocity perturbations* were always experienced as meaningful by the rowers, particularly as Simultaneously Diverging Experiences (SDE): 25% of the time (4 cycles out of a total of 16) by the international crew (Table 2) and 19.2% (5 cycles out of a total of 26) by the national crew (Table 3).

Our second finding pointed out that the *functional adaptations* were experienced in different ways: (a) Simultaneously and Similarly Experienced as Meaningless (SSE-L): 31.3% for the international crew vs. 11.5% for the national crew; (b) Simultaneously and Similarly as Meaningful (SSE-F): 25% for the international crew vs. 26.9% for the national crew; (c) Simultaneous Diverging Experiences (SDE): 6.3% for the international crew vs. 11.5% for the national crew; and (d) Not Simultaneously Experienced as Meaningful (NSEM): 12.5% for the international crew vs. 30.8% for the national crew. These findings highlight that for the most part the two rowers of the international crew simultaneously and similarly experienced functional adaptions. Conversely, the two rowers of the national crew alternated between simultaneous and not simultaneous meaningful experiences of their functional adaptations.

## Discussion

The main finding of our study was the close association between the stability in behavior and boat performance. In particular, boat velocity variability was associated with the variability in the interpersonal coordination and individual organization at kinematic and kinetic levels, which is in accordance with the literature (Soper and Hume, 2004; Hill and Fahrig, 2009; Nolte, 2011). However, it must be recalled that our study was only based on two cases; therefore, it is difficult to generalize the results and to run any statistical analysis.

From there, our aim was to focus on the cycles (for interpersonal coordination, individual organization and boat velocity measurements) outside of the confidence interval to investigate how rowers exploit degeneracy of the perceptual and motor systems when they coped with race constraints. Degeneracy property supported “*functional*” adaptations, because the behavior varied structurally while the boat's velocity remained stable. Conversely, behavioral variability was observed as “*perturbing*” when it leads boat's velocity outside the confidence interval. This can clearly be seen in the international men's pair at 400 and 540 s of the race, when drops in boat velocity (Figure 10) were associated with high variability in interpersonal coordination (Figures 2, 5, 6) and lived as simultaneously divergent experiences (Table 1); this observation led us to characterize these events as “behavioral perturbation.” Thus, the race constraints were associated with destabilized interpersonal coordination, called “behavioral perturbations” when the boat velocity decreased or “functional adaptations” when the boat velocity was maintained. This summary of our main findings suggests three aspects for in-depth discussion: (a) the functional vs. perturbing role of variability in interpersonal coordination; (b) the constraints that influence the interpersonal coordination dynamics in rowing, notably with respect to the roles given to the stroke (leader) and bow (follower) rowers; and (c) how the variability in interpersonal coordination was experienced and shared, particularly regarding whether the *functional adaptations* and *behavioral and velocity perturbations* were similarly experienced by the two rowers.

### Functional vs. perturbing variability in interpersonal coordination

The international crew exhibited 25 cycles outside of the confidence interval for the boat velocity and 8–10 cycles outside of the confidence interval for the behavioral parameters (i.e., *RMS* and *C*_*i*_ of the kinematic and kinetic parameters). The national crew showed 21 cycles outside of the confidence interval for the boat velocity and 14–18 cycles outside of it for the behavioral parameters. When the boat velocity and the behavioral parameters were considered together, Tables 2, 3 highlight that 16 cycles were outside of the confidence interval (accounting for 4.7% of the race time) for the international crew and 26 cycles were outside of it (accounting for 7.2% of the race time) for the national crew. Second, more than considering the boat velocity and the behavioral parameters *together*, the crucial issue was to determine whether the variability in interpersonal coordination could be functional for achieving the task-goal. Indeed, interpersonal coordination variability should not necessarily be construed as noise, detrimental to performance (Newell and Corcos, 1993; Newell et al., 2005, 2006). Nor should it always be viewed as error or deviation from an expert or theoretical model, constantly in need of correction in practitioners (Davids et al., 2006). Interpersonal coordination variability could instead be considered to exemplify the flexibility of rowers to respond to changes in dynamic performance constraints (Davids et al., 2003; Seifert and Davids, 2012; Seifert et al., 2016b). Thus, in line with our hypothesis that rowers might exploit the degeneracy property of perceptual and motor systems to cope with the race constraints (Seifert et al., 2014, 2016b), we have suggested that interpersonal coordination variability was functional when it was associated with performance stability. From there, we identified three scenarios depending on whether the behavioral variability was *functional* (i.e., without significant change in boat velocity) or *perturbing* (i.e., with significant change in boat velocity): *functional adaptation* (12 cycles for the international crew and 21 cycles for the national crew), *behavioral perturbation* (4 cycles for the international crew and 3 cycles for the national crew), and *velocity perturbation* (i.e., when only the boat velocity was affected without any behavioral modification, which concerned 2 cycles of the national crew). For 78% of the time, high behavioral variability was *functional* because it reflected adaptations to dynamical constraints in order to achieve the task-goal (e.g., the phenomenological data indicated that the rowers' behavioral adaptations were oriented toward acting on the boat direction or its velocity; see the last section for further discussion). However, 22% of the time, high behavioral variability was associated with a *perturbation* of the boat velocity. According to the magnitude and frequency of the inter-cycle variability of the stroke and bow rowers' respective motor organization, the high behavioral variability came from one rower (3% of the time; mainly the bow rower) or the two rowers simultaneously (14% of the time), or was not associated was the rowers' behavior (5% of time), confirming that interpersonal coordination in rowing is an important feature of performance (Hill, 2002; de Brouwer et al., 2013; Cuijpers et al., 2015). Our study showed that high variability in interpersonal coordination could occur at both kinematic and kinetic levels; however, the behavioral variability observed in the national crew may have been due to a lack of synchronization in force generation and a significantly greater difference in force impulse between the rowers (Figures 4, 5). The next section discusses how these functional adaptations or perturbations in interpersonal coordination can be explained by a set of interacting constraints, notably the role given to the stroke (leader) and bow (follower) rowers in the crew.

### Constraints influencing the coordination pattern dynamics in rowing

Our phenomenological data suggested that when rowers did not focus on themselves or their partners, they focused on various task and environmental constraints (e.g., waves, wind, other boats, changes in the river pathway, buoys indicating a certain distance from the end) that could be associated with a destabilization of their interpersonal coordination. As often observed in a range of cyclic movement tasks performed individually (in bimanual coordination, see Kelso, 1984; in postural regulation, see Bardy et al., 2002; in swimming, see Potdevin et al., 2006) or collectively (in the wrist-pendulum paradigm, see Schmidt et al., 1998; in postural regulation, see Varlet et al., 2011; in rowing, see Cuijpers et al., 2015), stroke frequency is a key task constraint that can act as a control parameter. In particular, Cuijpers et al. (2015) showed that when stroke frequency was increased, the synchronization between limbs and between individual actions was also increased. According to our phenomenological data, the rowers often focused on stroke frequency, boat velocity and boat direction, which might have constrained the coordination between the rowers, leading to *functional adaptation* or *perturbation* (Tables 2, 3).

Interestingly, these constraints interacted with another constraint theoretically given in advance: the role of each rower. As explained in the introduction, although it was expected that the stroke rower would lead the crew, while the bow rower followed the other's lead (Nolte, 2011), our results (Figures 8, 9) showed that the bow rower exhibited higher variability in his/her kinetic and kinematic parameters more often than the stroke rower. These results indicated that the stroke rower had to compensate or communicate with the bow rower to balance the interpersonal coordination (which was also reported by Lund et al., 2012). In fact, the phenomenological data of the national crew (cycle 13, Table 3) showed that the bow rower looked for information in his/her environment and even for instructions from the stroke rower, and sometimes asked the stroke rower to do a better job of driving the crew. The kinematic and kinetic gap between the stroke and bow rowers occurred very often for the national crew (Figure 9), which sometimes could not be self-regulated by the stroke rower. For instance, the stroke rower of the national crew turned back to communicate with the bow rower when she perceived dysfunction in the interpersonal coordination (cycle 22, Table 3). These types of behavior were observed by Sève et al. (2013) and confirmed that being coordinated with one's partner is a feature of expertise in cooperative contexts of performance (Hill, 2002; Baudouin and Hawkins, 2004). As observed in our study, several recent studies have shown that interpersonal coordination can be optimized by using miming and signaling strategies to communicate concerns to a partner (Sacheli et al., 2013; Candidi et al., 2015). The meaning of “rowing together” (Lund et al., 2012) through verbal and nonverbal communication confirms the importance given to both behavioral and phenomenological investigation (De Jaegher and Di Paolo, 2007). Indeed, because individuals participate in the “*generation of meaning through their bodies and action often engaging in transformational and not merely informational interactions*” (p. 39) (Di Paolo et al., 2011), the next section considers how the variability in interpersonal coordination (functional adaptation vs. perturbation) was experienced and shared (De Jaegher and Di Paolo, 2007).

### How the variability in interpersonal coordination was experienced and shared by the rowers

The combination of phenomenological and behavioral data in our study helped determine whether the *functional adaptations* or *behavioral and velocity perturbations* (identified from kinetic and kinematic data) were experienced by the two rowers (a) simultaneously or not simultaneously, (b) as meaningful or meaningless, and (c) as similar or diverging concerns.

Our first finding was that the *behavioral and velocity perturbations* were always experienced as meaningful by the rowers, particularly as Simultaneously and Diverging Experiences (SDE). This finding indicates that the rowers were able to spontaneously focus on information about boat direction and velocity, stroke frequency, other boats, buoys in the river, edges and turns in the river, all of which at times engaged their behavior differently and were associated with interpersonal coordination destabilization. The divergence in the two rowers' concerns also suggested that the predetermined roles of the stroke rower (i.e., given as leader) and bow rower (i.e., given as follower) were not always respected in the crew (as expected by the coach who paired the junior women rowers of the national crew). Thus, it can be hypothesized that such divergent concerns explain the destabilization in the interpersonal coordination and the boat velocity perturbations. However, it must be kept in mind that the destabilization in interpersonal coordination was associated with changes in boat velocity a few times; however, when boat velocity was perturbed, it never lasted for more than three consecutive cycles (according to Figures 8, 9).

Our second finding was that the *functional adaptations* in the international crew were mainly experienced simultaneously and similarly, sometimes as meaningless and sometimes as meaningful. This emphasizes that at the international level, the rowers were able to exhibit adaptive variability in their behavior (i.e., individual kinetic or kinematic data outside of the confidence interval) and experience it as meaningless (as already underlined by R'Kiouak et al., 2016). In addition, when the rowers experienced a destabilization in their behavior and/or interpersonal coordination as meaningful, they seemed to do so mainly simultaneously and similarly. According to De Jaegher and Di Paolo (2007), this highlights how international rowers can coordinate their experience through interactions and not just physical manifestations. Indeed, as noted by Lund et al. (2012), many times the international rowers both performed and felt the “*joint rhythm*,” suggesting that they were able to feel their partner's actions through the boat velocity variations in order to minimize them (Millar et al., 2013).

Conversely, the two rowers of the national crew alternated between simultaneous and not simultaneous meaningful experiences of their functional adaptions. This finding suggests that a lack of shared experiences would explain why the national crew exhibited more cycles for which the kinetic and/or kinematic data were outside of the confidence interval. Once again, this can be explained by the asymmetric relationship expected by the coach due to the greater experience of the stroke rower in the national crew. As noted by Millar et al. (2013), rowers can alternatingly focus on themselves, their partners and boat behavior, suggesting that sharing simultaneous and similar experiences and behaviors is a highly complex coordination process.

In conclusion, the investigation of how rowers coordinate their behavior and experience helped explain how high variability in interpersonal coordination can result in being either functional or perturbing; either meaningful or meaningless; and either similar or diverging. Degeneracy property of perceptual and motor systems can help to understand how structural variability of the behavior could be either “*functional*” (when associated to functional stability, i.e., stability of the boat velocity) or “*perturbing*” (when associated to significant change of the boat velocity). However, although boat velocity variations between cycles appeared as the main contributor to assess rowing performance, using only this parameter to assess the performance outcome might be a limitation of this study. Additional measure of boat heading orientation might help to understand adjustment onto the velocity. Phenomenological data helped to mitigate that limitation by gathering information about the perceived purpose of the coordination changes by the rowers. Indeed, by combining phenomenological and behavioral data, these two case studies showed how constraints—not manipulated by an experimenter but emerging from the ecological context of a race—can be associated with functional adaptations or behavioral perturbations of interpersonal coordination. As already advanced by Millar et al. (2013), our findings suggest that high expertise implies a better feel for one's partner through the boat, which might reflect a greater appropriation of boat behavior. Nevertheless, this interpretation must be further explored with bigger samples of crews.

## Author contributions

Contribution of the authors are as follows: Conception or design of the work: JB, JL, AN, JS. Acquisition of the data: JB, JL, AN, JS. Analysis of the data: JB, DA, LS, JL, JS. Interpretation of data for the work: JB, DA, LS, JL, JS, RT. Drafting the work or revising it critically for important intellectual content: JB, DA, LS, JL, JS, RT. Final approval of the version to be published: JB, DA, LS, JL, JS, RT. Agreement to be accountable for all aspects of the work in ensuring that questions related to the accuracy or integrity of any part of the work are appropriately investigated and resolved.

### Conflict of interest statement

The authors declare that the research was conducted in the absence of any commercial or financial relationships that could be construed as a potential conflict of interest.
